# Lymphocytic Choriomeningitis Virus Infections among American Indians

**DOI:** 10.3201/eid1902.120888

**Published:** 2013-02

**Authors:** Barbara Knust, Robert C. Holman, John Redd, Jason M. Mehal, Steven M. Grube, Adam MacNeil, James Cheek, Pierre E. Rollin

**Affiliations:** Author affiliations: Centers for Disease Control and Prevention, Atlanta, Georgia, USA (B. Knust, R.C. Holman, J.M. Mehal, S.M. Grube, A. MacNeil, P.E. Rollin);; Indian Health Service, Albuquerque, New Mexico, USA (J. Redd, J. Cheek)

**Keywords:** lymphocytic choriomeningitis virus, LCMV, viruses, lymphocytic choriomeningitis, American Indians, incidence, electronic diagnostic codes, chart review, International Classification of Diseases, ICD-9

**To the Editor:** Lymphocytic choriomeningitis virus (LCMV) is a rodent-borne pathogen that causes a spectrum of disease in humans, ranging from self-limiting meningoencephalitis to congenital birth defects to severe disseminated illness in organ transplant recipients (*1*). It is not known how frequently cases of LCMV infection are diagnosed in the United States. We performed a retrospective case analysis of American Indian and Alaska Native (AI/AN) patients treated within the Indian Health Service (IHS) health care system who had an LCMV-associated diagnosis defined by the International Classification of Diseases, 9th Revision, Clinical Modification (ICD-9-CM). Our goal was to estimate the incidence of LCMV-associated aseptic meningitis and encephalitis diagnosed within a well-defined population.

Inpatient and outpatient visit data from fiscal years 2001–09 were obtained from the IHS National Patient Information Reporting System (*2*). For each fiscal year, records for AI/AN patients with at least 1 inpatient or outpatient visit within the year that listed the ICD-9-CM code 049.0 (lymphocytic choriomeningitis and meningoencephalitis) were selected (*3*,*4*). A subset of medical records was reviewed for patients with the diagnosis code of interest in the IHS Southwest and Southern Plains regions. Health care facilities were located in Arizona, New Mexico, and Oklahoma, USA. Suspected LCMV infection was defined as a diagnosis of meningitis, choriomeningitis, encephalitis, or meningoencephalitis not explained by another etiologic agent. A confirmed case of LCMV infection required laboratory detection of antibodies, virus antigen, or virus. A patient with no evidence of suspected LCMV infection in his or her medical record was not considered to have a case of LCMV infection.

Annual population denominators were determined by using annual IHS Southwest and Southern Plains region user populations, which includes all registered AI/ANs who received IHS-funded health care at least once during the previous 3 years (*5*). The annual average incidence rate for diagnosed cases of infection with LCMV was determined. Rates were also determined for viral meningitis not otherwise specified (ICD-9-CM code 047.9); for unspecified causes of encephalitis, myelitis, or encephalomyelitis (323.9); and for unspecified non-arthropod–borne viral diseases of the central nervous system or viral encephalitis not otherwise specified (049.9) (*3*). Annual numbers were calculated on a patient basis, whereby the first time a diagnosis was coded for a given patient during each fiscal year was counted.

Twenty-six AI/AN patients received the diagnosis code of 049.0 during fiscal years 2001–09. Of these patients, 16 received the diagnosis in the Southwest or Southern Plains regions. Fourteen available medical charts from these 2 regions were reviewed, and 4 patients were classified as having signs and symptoms consistent with suspected LCMV infection (Table), although no patients were confirmed by diagnostic testing as having LCMV infection. All 4 suspected cases of LCMV infection were in women (age range 16–43 years) who had a 1–2-day history of headache, nausea, and vomiting. Photophobia and neck or back pain were present in patients 1–3. Cerebrospinal fluid from patients 2–4 had increased leukocyte counts that were lymphocytic.

**Table:** Clinical features and diagnoses for 14 American Indian patients with an ICD-9-CM diagnosis code of 049.0 (lymphocytic choriomeningitis), Southwest and Southern Plains IHS regions, United States, fiscal years, 2001–09*

Patient no.	CNS signs and symptoms	Lymphocytes in CSF	Diagnosis	Status
1	Yes	Yes	Viral meningitis	Suspected LCM
2	Yes	Yes	Lymphocytic meningitis	Suspected LCM
3	Yes	Yes	Lymphocytic meningitis	Suspected LCM
4	Yes	Yes	Lymphocytic meningitis	Suspected LCM
5	Yes	Yes	Tuberculosis meningitis	Miscoded
6	Yes	No	Lyme encephalitis	Miscoded
7	No	No	Lymphocele	Miscoded
8	No	No	Lymphocytic cholitis	Miscoded
9	No	No	Lymphocytic leukemia	Miscoded
10	No	No	Lymphocytic leukemia	Miscoded
11	No	No	Atypical lymphocytes	Miscoded
12	No	No	Reactive lymphocytes	Miscoded
13	No	No	Charcot foot anomaly	Miscoded
14	No	No	Tuberculosis	Miscoded

*Diagnosis was the first listed or most clinically relevant written diagnosis from the medical record. Patient status (confirmed or suspected lymphocytic choriomeningitis [LCM] or miscode) was determined by the chart reviewer on the basis of the criteria. ICD-9-CM, International Classification of Diseases, 9th Revision, Clinical Modification; IHS, Indian Health Service; CNS, central nervous system; CSF, cerebrospinal fluid.

The diagnoses of the 10 remaining patients were classified as miscodes because the written diagnoses in the charts did not mention lymphocytic meningitis. Two patients had central nervous system disease (Lyme encephalitis and tuberculosis meningitis) although an etiologic agent was confirmed that was not LCMV. Additional diagnoses mistakenly coded are shown in the Table.

Among the 4 patients identified as having clinical signs and symptoms of suspected LCMV infection in the Southwest and Southern Plains regions during fiscal years 2001–09, the average annual incidence rate was estimated to be 0.06 cases/100,000 persons. In the same population, viral meningitis not otherwise specified was reported for 971 patients (incidence rate 13.69 cases/100,000 persons/year). Unspecified causes of encephalitis, myelitis, or encephalomyelitis were diagnosed for 444 patients (incidence rate 6.26 cases/100,000 persons), and unspecified non-arthropod–borne viral diseases of the central nervous system or viral encephalitis not otherwise specified were diagnosed for 56 patients (incidence rate 0.78 cases/100,000 persons).

Using a population-based analysis of diagnoses for patients who visited the IHS system at least 1 time as an inpatient or outpatient, we found that LCMV-associated aseptic meningitis and encephalitis were infrequently diagnosed and that confirmatory testing was not conducted. We also found that the code for LCMV was used incorrectly for several patients, probably because the virus is named ambiguously, and LCMV could be mistaken for other unrelated diseases with a lympho prefix. In addition, the definition for the code does not specify LCMV infection. We recommend that description of the ICD-9-CM and ICD-10 codes should be adjusted accordingly to clarify and reduce coding errors.

LCMV infection is considered to be underdiagnosed (*6*,*7*), although the virus was not detected in several studies that prospectively tested for LCMV in large numbers (>90) of clinical encephalitis or meningitis patients (*8*–*10*). Despite infrequently causing aseptic meningitis or mild disease in immunocompetent persons, LCMV has the potential to cause severe disease and should be considered for patients with a compatible illness and potential rodent exposure.
